# Surface plasmons induce topological transition in graphene/α-MoO_3_ heterostructures

**DOI:** 10.1038/s41467-022-31477-z

**Published:** 2022-06-28

**Authors:** Francesco L. Ruta, Brian S. Y. Kim, Zhiyuan Sun, Daniel J. Rizzo, Alexander S. McLeod, Anjaly Rajendran, Song Liu, Andrew J. Millis, James C. Hone, D. N. Basov

**Affiliations:** 1 Department of Physics, Columbia University, New York, NY USA; 2 Department of Applied Physics and Applied Mathematics, Columbia University, New York, NY USA; 3 Department of Mechanical Engineering, Columbia University, New York, NY USA; 4 Center for Computational Quantum Physics, Flatiron Institute, New York, NY USA

**Keywords:** Polaritons, Optical properties and devices, Metamaterials, Sub-wavelength optics, Scanning probe microscopy

## Abstract

Polaritons in hyperbolic van der Waals materials—where principal axes have permittivities of opposite signs—are light-matter modes with unique properties and promising applications. Isofrequency contours of hyperbolic polaritons may undergo topological transitions from open hyperbolas to closed ellipse-like curves, prompting an abrupt change in physical properties. Electronically-tunable topological transitions are especially desirable for future integrated technologies but have yet to be demonstrated. In this work, we present a doping-induced topological transition effected by plasmon-phonon hybridization in graphene/α-MoO_3_ heterostructures. Scanning near-field optical microscopy was used to image hybrid polaritons in graphene/α-MoO_3_. We demonstrate the topological transition and characterize hybrid modes, which can be tuned from surface waves to bulk waveguide modes, traversing an exceptional point arising from the anisotropic plasmon-phonon coupling. Graphene/α-MoO_3_ heterostructures offer the possibility to explore dynamical topological transitions and directional coupling that could inspire new nanophotonic and quantum devices.

## Abstract (web summary)

Hyperbolic phonon polaritons – mixed states of photons and anisotropic lattice vibrations – offer appealing properties for nanophotonic applications. Here, the authors show that the plasmon-phonon hybridization upon electronic doping in graphene/α-MoO3 heterostructures can induce topological transitions of the polariton wavefront.

## Introduction

Hyperbolic optics first rose to prominence with the development of hyperbolic metamaterials constructed by periodically alternating metallic and dielectric media^1^. The term “hyperbolic” refers to the shape of the isofrequency surface, which is hyperboloidal rather than ellipsoidal like in common anisotropic media, because of the opposite signs of the real dielectric permittivities of metallic and dielectric media. Hyperbolic isofrequency surfaces give rise to an increased photonic density of states, which can help engineer spontaneous^2^ and thermal^3^ emission. Furthermore, the unique properties of hyperbolic media have allowed for the development of subwavelength imaging^4^, polarization converters^5^, and negative refraction^6^, among many other technologies.

In the past decade, natural van der Waals materials including hexagonal boron nitride (h-BN)^7–9^ and α-phase molybdenum trioxide (α-MoO_3_)^10,11^ have been found to exhibit hyperbolicity around anisotropic phonon resonances that drive permittivity to negative values along one crystal axis or plane^12–14^. Van der Waals crystals display relatively weak phonon damping, enabling propagating phonon polaritons that behave like waveguide modes with deeply subdiffractional wavelengths^15^. Phonon polaritons in the two reststrahlen bands of uniaxial h-BN are out-of-plane hyperbolic, meaning that the out-of-plane optic axis and its normal plane have opposite-signed permittivities. h-BN polaritons can propagate along any in-plane direction^7–9^. On the other hand, α-MoO_3_ is biaxial with three infrared reststrahlen bands. The upper band hosts out-of-plane hyperbolic modes (958–1010 cm^−1^$:{\varepsilon }_{x} \, > \, 0,{\varepsilon }_{y} \, > \, 0,{\varepsilon }_{z} < 0$), while the middle (820–972 cm^−1^$:{\varepsilon }_{x} < 0,{\varepsilon }_{y} \, > \, 0,{\varepsilon }_{z} \, > \, 0$) and lower (545–851 cm^−1^: ${\varepsilon }_{x} \, > \, 0,{\varepsilon }_{y} < 0,{\varepsilon }_{z} \, > \, 0$) bands have in-plane directions with opposite-signed permittivities and support in-plane hyperbolic modes with directional propagation and hyperbolic wavefronts^10,11^.

Integrated nanophotonics requires dynamical tuning of hyperbolic polaritons, but this is challenging since hyperbolicity in the two most widely studied systems (h-BN and α-MoO_3_) is rooted in crystal structure. Heterostructuring and hybridizing hyperbolic modes with other modes can modify their properties^16–22^, but adjusting tuning parameters typically requires altering device design. With α-MoO_3_, for example, in-plane hyperbolic phonon polaritons can be modified by fabricating double-layer structures with a predefined twist angle as the tuning parameter^16–19^. The isofrequency contour (IFC) characterizing the propagation-direction-dependent momenta of in-plane hyperbolic modes can be tuned through a topological transition^23^ from an open hyperbola to a closed curve by sweeping twist angle. On the other hand, dynamical tuning of h-BN phonon polaritons has been demonstrated by means of hybridization with graphene surface plasmons^20^. Surface plasmon-phonon polariton (SP^3^) and hyperbolic plasmon-phonon polariton (HP^3^) hybrid modes have gate-tunable dispersions enabled by the carrier-density-dependent Fermi energy ${E}_{F}$ of graphene^24^. However, graphene/h-BN admits only modest electronic tunability without topological transitions.

Here, we present a graphene on α-MoO_3_ heterostructure that can be tuned electronically through a topological transition as evidenced by direct imaging of propagating polaritons. At charge neutrality, graphene does not host plasmons and the IFC is a hyperbola. Upon electronic doping, the IFC transitions to a closed curve as graphene surface plasmons hybridize with α-MoO_3_ hyperbolic phonon polaritons. We demonstrate the topological transition of the polariton wavefront experimentally and tune momenta of hybrid modes by modifying E_F_ and laser frequency $\omega$. Furthermore, calculations corroborated by experiments reveal that, by rotating the polaritonic wavevector, the hybrid mode characterized by the closed IFC is tuned from HP^3^ to SP^3^ as its plasmon-phonon coupling is modified. Graphene/α-MoO_3_ is thus a platform for studying the effect of anisotropic plasmon-phonon coupling on properties of hybrid hyperbolic polaritons. In particular, we identify an exceptional point in the direction-dependent polariton dispersion.

## Results and discussion

### Hybrid polariton theory

**Fig. 1:**
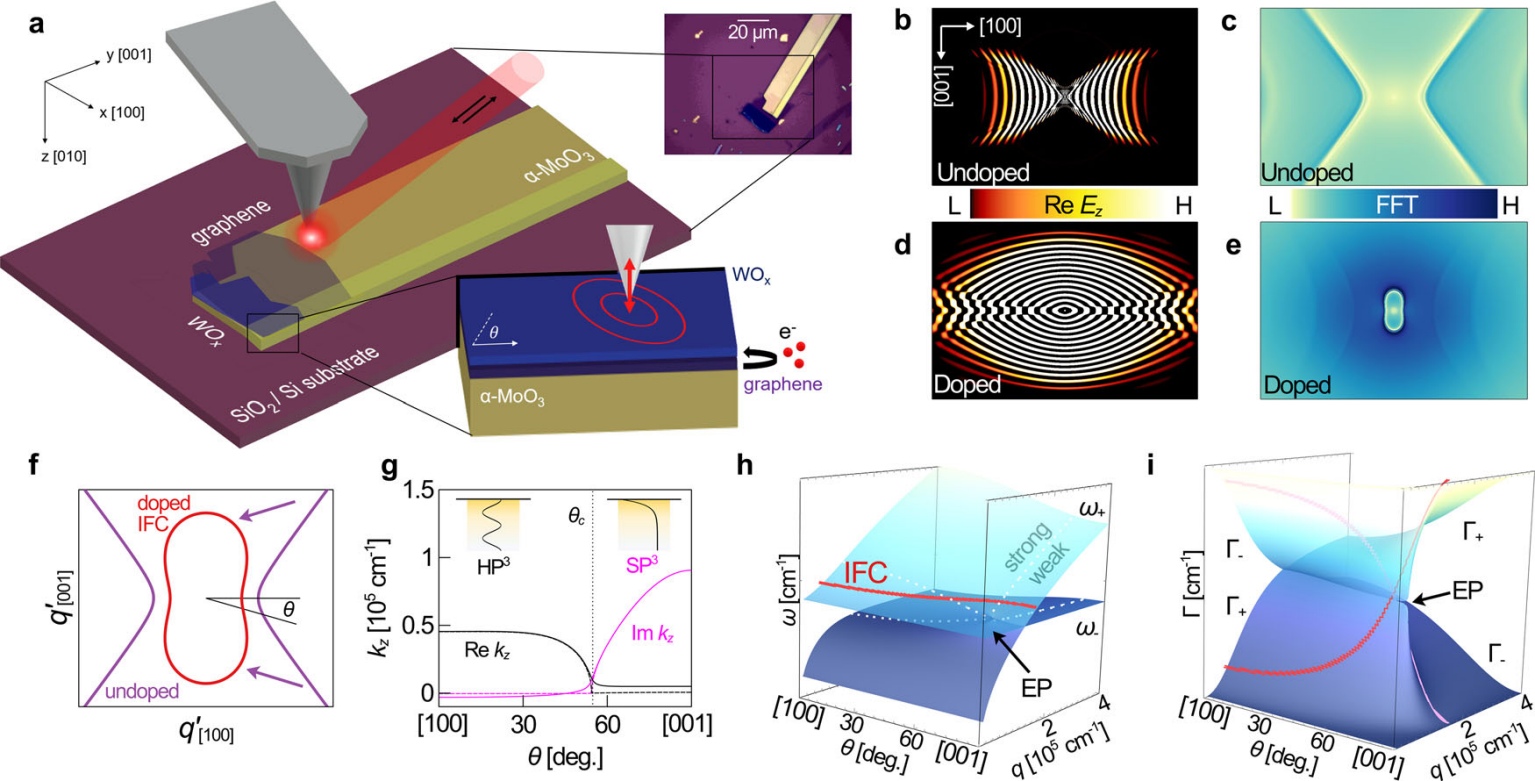
Doping-induced topological transition by plasmon-phonon hybridization. **a** Graphene/α-MoO_3_ device schematic in a scattering-type scanning near-field optical microscope (s-SNOM). Inset shows an optical microscope image of a sample. Graphene doping is achieved via charge transfer with WO_x_. The s-SNOM tip acts as a dipole antenna, launching polaritons in all permitted directions with in-plane angle $\theta$. Without doping, there is no propagation along [001] direction and the, **b** wavefront and, **c** isofrequency contour (IFC) are hyperbolic. Upon doping, propagation is allowed along the [001] direction and the, **d** wavefront and, **e** isofrequency contour of the first-order mode are closed curves. **f** Semi-analytical IFC undergoing topological transition upon doping. **g** Along the [100] direction, polaritons have out-of-plane propagation constant k_z_ with Re k_z_ » Im k_z_ such that the out-of-plane electric field is oscillatory like a waveguide mode (HP^3^, left inset). Along the [001], Im k_z_ » Re k_z_ and polaritons are surface waves with decaying electric field (SP^3^, right inset). **h** Plasmon mode splitting (${\omega }_{{pl}}\to {\omega }_{+}\& \,{\omega }_{-}$) caused by the [100] α-MoO_3_ phonon as a function of *q* and *θ*. The plot reveals an exceptional point (EP). The doped IFC from **f** is a curve on the ${\omega }_{+}$ surface (red line). The IFC passes about the EP, indicating a transition from strong to weak coupling. White dotted lines are guides to the eye. **i** Scattering rates Γ_+_& Γ_−_ of *ω*_*+*_ & *ω*_−_ modes are anticorrelated (compare red and pink lines).

In this work, we focus on frequencies in the middle reststrahlen band of α-MoO_3_ (820–972 cm^−1^) where the topological transition occurs. Finite difference time domain simulations (Methods) show that, without doping, graphene is devoid of plasmonic response and the heterostructure is optically equivalent to bare α-MoO_3_ (neglecting losses in charge-neutral graphene). In the cleavage plane of the [100] (x) and [001] (y) crystal axes (Fig. 1a), the phonon polariton wavefront is a hyperbola (Fig. 1b). The IFC, related to the wavefront by a Fourier transform, is likewise a hyperbola (Fig. 1c). Multiple hyperbolas appear: corresponding to higher-order, short wavelength hyperbolic modes. Upon doping the graphene to appreciable ${E}_{F}$, the wavefront becomes a closed curve with anisotropic polariton wavelengths as a result of plasmon-phonon hybridization (Fig. 1d). The first-order IFC undergoes a topological transition to a closed peanut shape (Fig. 1e).

The direction-dependent dispersion $q^{\prime} (\omega ,\theta )$ and dissipation $q^{\prime\prime} (\omega ,\theta )$ of hybrid modes in graphene with optical conductivity ${\sigma }_{g}$ on a biaxial α-MoO_3_ slab of thickness $d$ at arbitrary frequency $\omega$ can be computed by solving Equation S5 (Supplementary Note 1). Figure 1f shows that calculated IFCs at ${E}_{F}=0$ eV and 0.4 eV in the middle reststrahlen band are consistent with simulated IFCs in Fig. 1c, e, respectively. The angle $\theta$ specifies the in-plane propagation direction with respect to the [100] direction. The out-of-plane propagation constant inside α-MoO_3_ is ${k}_{z}\approx \pm q\sqrt{-{\varepsilon }_{\parallel }/{\varepsilon }_{z}}$ where ${\varepsilon }_{\parallel }={\varepsilon }_{x}{{{\cos }}}^{2}\theta +{\varepsilon }_{y}{{{\sin }}}^{2}\theta$. In the doped structure, the wave launched by the tip is a surface wave (SP^3^: Im ${k}_{z}$ ≫ Re ${k}_{z}$) along the [001] direction; and a waveguide mode (HP^3^: Re ${k}_{z}$ ≫ Im ${k}_{z}$) along the [100] direction. Along intermediate directions (Fig. 1g), the polariton is an HP^3^ up to the open angle ${\theta }_{c}={{\arctan }}\sqrt{-{{{{{\rm{Re}}}}}}\,{\varepsilon }_{x}/{{{{{\rm{R}}}}}}{{{{{\rm{e}}}}}}\,{\varepsilon }_{y}}$, i.e., the asymptote of the undoped hyperbola. Above ${\theta }_{c}$, it is an SP^3^. Note that HP^3^ and SP^3^ in this work are only defined by their k_z_. At ${\theta }_{c}$, both real and imaginary components of ${k}_{z}$ drop precipitously. Neglecting in-plane losses (dashed lines), ${k}_{z}=0$ at ${\theta }_{c}$: the polariton is an ideal plane wave propagating in-plane with finite momentum.

Along the [001] direction in the middle reststrahlen band, the α-MoO_3_ phonon is absent and the plasmon-phonon coupling strength must be zero. Along the [100] direction, graphene plasmons and α-MoO_3_ phonons hybridize due to strong coupling; that is, the plasmon mode splits into plasmon-phonon polariton branches above and below the a-axis transverse optical (TO) phonon frequency ${\omega }_{{TO}}^{a}$. Tuning the in-plane propagation angle $\theta$ must then modify the plasmon-phonon coupling, which transitions at some point between strong and weak coupling regimes. Prior work^25^ has recognized that the transition occurs at an exceptional point (EP), above which plasmons experience Rabi splitting from strong coupling to phonons. Below the EP, there is no mode splitting: plasmons and phonons are weakly coupled in a manner akin to electromagnetically-induced transparency.

We now show quantitatively that tuning $\theta$ can be expected to modify plasmon-phonon coupling. Consider two-dimensional α-MoO_3_ with high-frequency permittivities set to unity. The total dielectric function of Drude-like graphene with electron coupling to in-plane hyperbolic phonons modulated by the quantity $\alpha (q,\theta )\equiv \frac{{qd}}{2}{{{\cos }}}^{2}\theta$ is then:

1 $${\varepsilon }_{2D}\left(\omega ,q,\theta \right)=1-\frac{{\omega }_{{pl}}^{2}\left(q\right)}{{\omega }^{2}+i{\omega \gamma }_{g}}+\alpha \,(q,\theta )\,\frac{{\omega }_{{LO}}^{2}-{\omega }_{{TO}}^{2}}{{\omega }_{{TO}}^{2}-{\omega }^{2}-i\omega \gamma },$$

where ${\omega }_{{pl}}^{2}(q)\equiv q\left|{E}_{F}\right|{e}^{2}/2\pi {\hslash }^{2}{\varepsilon }_{0}$ and ${\gamma }_{g}$ and $\gamma$ are the plasmon and phonon scattering rates, respectively (Supplementary Note 2). The real and imaginary roots of Eq. 1 are plotted in Fig. 1h, i, respectively. Larger $\alpha$ near the [100] direction split plasmon modes at ${\omega }_{{pl}}$ into two branches above and below ${\omega }_{{TO}}^{a}$, labeled ${\omega }_{+}$ and ${\omega }_{-}$, respectively (Fig. 1h). ${\omega }_{+}$ and ${\omega }_{-}$ have anticorrelated scattering rates, ${\Gamma }_{+}$ and ${\Gamma }_{-}$ (Fig. 1i). Splitting is reduced as $\theta$ rotates toward the [001] direction until reaching a complex degeneracy, beyond which ${\omega }_{+}$ and ${\omega }_{-}$ coalesce and ${\Gamma }_{+}$ and ${\Gamma }_{-}$ split. This degeneracy is in fact the exceptional point discussed previously that marks the crossover from strong to weak coupling. The doped IFC from Fig. 1f is a curve (solid red line) on the ${\omega }_{+}$ surface, passing about the EP and traversing from strong to weak coupling (white dotted lines) in the two-dimensional limit as the mode evolves from HP^3^ to SP^3^ in the finite slab.

### Nano-imaging and tunability

A scattering-type scanning near-field optical microscope (s-SNOM) was used to image polaritons and evaluate their energy-momentum ($\omega ,q$) dispersions (Methods). An s-SNOM is a tapping-mode atomic force microscope with a metallized tip illuminated by laser light. The sharp metallic s-SNOM tip launches propagating polaritons (Fig. 1a) that reflect off of sample edges and defects. The launched and reflected waves interfere and form standing waves, which are imaged by the scanning probe. Polariton momentum and dissipation, quantified by real and imaginary $q={q}^{\prime}+{iq}^{\prime\prime}$, respectively, were extracted from observed wavelengths $\lambda =\pi /q^{\prime}$ and propagation lengths $L=1/q^{\prime\prime}$ (Methods). We used oxidized tungsten diselenide (WO_x_) to dope graphene^26^ by a charge transfer process (Methods). Doping graphene using thin high-work-function materials like WO_x_ or α-RuCl_3_ has proven to be nondetrimental to s-SNOM imaging^27,28^. Furthermore, by stacking either monolayer or bilayer tungsten diselenide (WSe_2_) prior to oxidation, we can tune the ${E}_{F}$ of graphene on α-MoO_3_ between ~0.60 eV and 0.45 eV, respectively. Doping levels were determined by fitting ($\omega ,q$) data to calculated dispersions with graphene Fermi energy as a free parameter, and were corroborated by Raman spectroscopy (Supplementary Fig. 2, Supplementary Note 3).

**Fig. 2:**
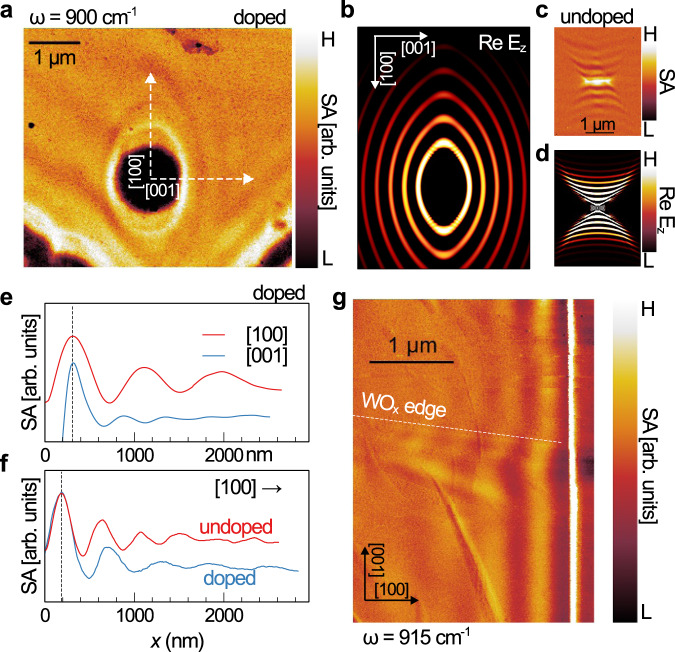
Near-field infrared imaging of hybrid plasmon-phonon modes. **a**, **b** Near-field amplitude image of a WO_x_/graphene/α-MoO_3_ heterostructure with ~0.60 eV hole doping in graphene from charge transfer with the WO_x_ monolayer. The experimental polariton wavefront displays the characteristic elliptical shape from finite-difference time-domain simulations shown in panel **b** to the right. **c** Near-field amplitude image and, **d** simulated wavefront of a defect in bare α-MoO_3_ launching hyperbolic wavefronts—in contrast to the closed wavefront in **a** where [001] modes appear after the topological transition caused by plasmon-phonon hybridization. **e** Line profiles extracted along the white dashed lines in **a** showing both [100] and [001] modes. Black dashed lines align the first peaks. **f** [100] Line profiles extracted from either side of the WO_x_ edge (white dashed line) in **g**. Wavelengths of [100] modes increase as a result of plasmon-phonon hybridization upon doping.

In Fig. 2, we image polaritons in various graphene/α-MoO_3_ heterostructures with and without graphene doping. In Fig. 2a, a circular void in highly-doped graphene (Supplementary Figure 3) produces a closed wavefront with the characteristic elliptical shape of hybrid plasmon-phonon polaritons. [100] and [001] modes as well as modes at intermediate angles are visible. The simulated Re ${E}_{z}$ wavefront (Fig. 2b) is consistent with the experimental wavefront. For reference, we also obtained a near-field amplitude image (Fig. 2c) and simulated wavefront (Fig. 2d) on bare α-MoO_3_. We observe the hyperbolic wavefront and absence of [001] modes consistent with previous reports^10,11^. In Fig. 2e, we show line profiles extracted from the white dashed lines in Fig. 2a. The [100] and [001] modes have anisotropic wavelengths (compare the red and blue lines). Additionally, in Fig. 2f, we show [100] line profiles from either side of the WO_x_ edge in Fig. 2g (white dashed line) showing an increase in polariton wavelength upon doping. Fringes bend at the boundary between doped and undoped regions. The measured polariton wavelength on graphene/α-MoO_3_ without WO_x_ is consistent with low to no doping, indicating that charge transfer is negligible between graphene and α-MoO_3_ (Supplementary Fig. 4, Supplementary Note 3).

**Fig. 3:**
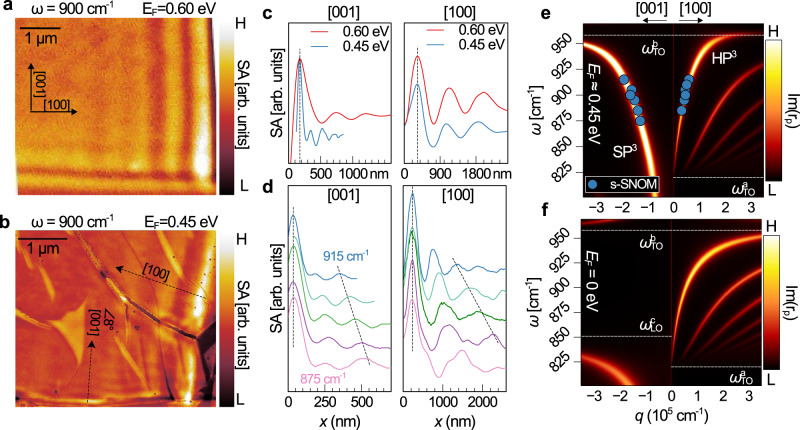
Tuning hybrid modes by WO_x_/WSe_2_ layer number and laser frequency. Near-field amplitude images of, **a** WOx/graphene/α-MoO_3_ with ~0.60 eV hole doping and, **b** WO_x_/1L-WSe_2_/graphene/α-MoO_3_ with ~0.45 eV hole doping. [100] and [001] modes are visibly less anisotropic in the more highly doped sample. **c** Near-field line profiles showing that increasing doping level substantially increases the wavelength of [001] modes, but only marginally increases [100] mode wavelengths. Black dashed lines align the first peaks. **d** Line profiles extracted from the sample in **b** excited at different laser frequencies from 875–915 cm^−1^. Note [001] modes in **b** are reflected from a physical boundary of the WO_x_/graphene layer at 8° from the [001], which is practically equivalent to the [001] direction (Supplementary Fig. 6). **e** Momenta from fitting profiles in **d** are consistent with the dispersion calculated from the imaginary part of the *p*-polarized reflection coefficient. ${\omega }_{{TO}({LO})}^{j}$ are TO(LO) frequencies of $j$-axis phonons: values plotted as white dashed lines. Note that there is a splitting at ${\omega }_{{TO}}^{b}$ from the upper Reststrahlen band phonon not considered in Eq. 1. **f** Calculated dispersion of phonon polaritons in bare α-MoO_3_ showing the absence of [001] modes for $\omega$=875–915 cm^−1^.

Near-field amplitude images at $\omega$=900 cm^−1^ on monolayer and bilayer WSe_2_ samples with *E*_*F*_ ≈ 0.60 eV and *E*_*F*_ ≈ 0.45 eV are shown in Fig. 3a, b, respectively. The [001] direction, which prohibits polariton propagation in bare α-MoO_3_ at this frequency, now hosts an SP^3^. We observe a significant tunability of the [001] mode by modifying ${E}_{F}$ (Fig. 3c), while the wavelength of the [100] mode changes only slightly (compare blue and red lines). The degree of anisotropy between orthogonal directions can thus be tuned dramatically by modifying ${E}_{F}$. Both [100] and [001] modes disperse as $\omega$ is tuned from 875–915 cm^−1^ (Fig. 3d), in agreement with the calculated dispersion (Fig. 3e). Note that SP^3^ modes merge with the first-order hyperbolic phonon mode of the lower reststrahlen band below ${\omega }_{{LO}}^{c}$, becoming HP^3^ modes and distinguishing them from pure plasmons. We remark that [001] modes in Fig. 3b are reflected from a physical boundary of the WO_x_/graphene layer that is 8° from the [001] direction (Supplementary Fig. 5). In Supplementary Fig. 6, we show that 8° yields a negligible correction to the dispersion calculation and we consider these modes to be effectively along the [001]. In Fig. 3e, the [100] HP^3^ has higher-order branches. Some extra fringes likely corresponding to higher-order modes are visible in 875 cm^−1^ and 885 cm^−1^ profiles (pink and purple in Fig. 3d). For reference, we also plot the polariton dispersion on bare α-MoO_3_ (Fig. 3f). Modes are absent along the [001] direction for $\omega$ = 875–915 cm^−1^.

### Topology and dissipation

**Fig. 4:**
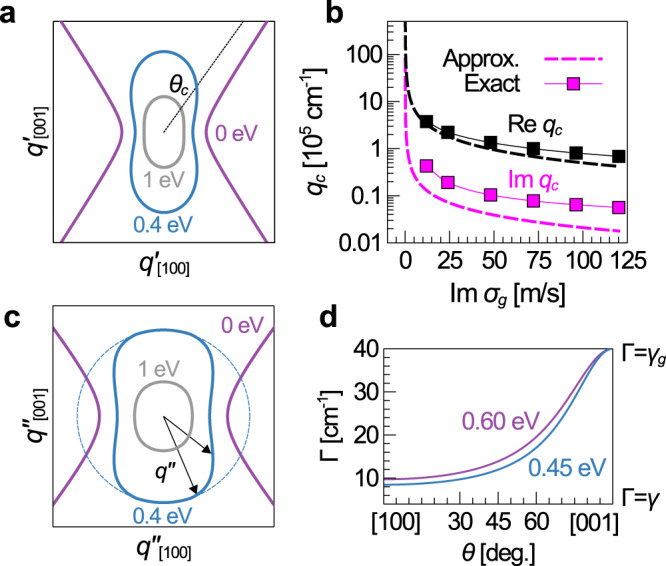
Directional dissipation and topological transition point. **a** IFC evolves with graphene Fermi energy from hyperboloid (0 eV) to peanut shape (0.4 eV) to extended circle (1 eV). At intermediate doping, the IFC inflects around the open angle ${\theta }_{c}$ (black dashed line) which marks the HP^3^ to SP^3^ transformation. **b** Dependence of momentum ${q}_{c}$ at ${\theta }_{c}$ on the graphene conductivity modulated by the Fermi energy. The topological transition occurs when ${q}_{c}$ becomes finite upon deviation from charge neutrality in an approximate analytical model (dashed lines). Full electrodynamics calculations (squares) support the trend up to low conductivities**. c** Loss contour at 0.4 eV shows $q\hbox{''}$ decreases in hybrid mode (solid blue) relative to uncoupled graphene (dashed blue) as the wavevector (black arrow) rotates away from the [001] direction. The undoped loss contour (purple) diverges at ${\theta }_{c}$, beyond which no mode exists. **d** Along the [001] direction, the hybrid mode scattering rate $\Gamma ={v}_{g}q\hbox{''}$ is equal to the graphene scattering rate ${\gamma }_{g}$. Along the [100] direction, $\Gamma$ is slightly larger than the phonon scattering rate $\gamma$ and can be tuned slightly by moderate doping.

We now expound on the exact topological transition point in graphene/α-MoO_3_ and the role of dissipation. Figure 4a shows the IFC evolving with doping from hyperbola to peanut to extended circle. The peanut-shaped IFC (blue contour in Fig. 4a) goes from concave to convex shape around the open angle ${\theta }_{c}$. Divergence of the wavevector ${q}_{c}$ at ${\theta }_{c}$ marks the topological transition, assuming no other $q$ diverges first, since bounded and unbounded curves cannot be homeomorphic. ${q}_{c}$ increases as the graphene conductivity decreases (Fig. 4b), diverging at ${\sigma }_{g}=0$ in an approximate analytical equation neglecting in-plane losses (dashed lines):

2 $$\frac{{q}_{c}}{{q}_{p}}=\frac{1}{4}\left(1-\zeta +\sqrt{{\left(1-\zeta \right)}^{2}+8\zeta }\right),\zeta \equiv \frac{2{\varepsilon }_{z}}{d{q}_{p}},$$

where ${q}_{p}\equiv i\omega /2\pi {\sigma }_{g}$ (Supplementary Note 1). Upon incremental doping away from zero conductivity, the IFC changes topology as momenta above ${\theta }_{c}$ gain large but finite values. In this picture, the topological transition point is exactly at ${\sigma }_{g}=0$ since even marginally-conducting IFCs will be bounded and only the hyperbola at ${\sigma }_{g}=0$ will be unbounded. Full numerical calculations (squares in Fig. 4b, Equation S5) support this trend up to low conductivities.

In practice, [001] modes will not be observable until appreciable Fermi energy ${E}_{F}$ with modest ${\sigma }_{g}$ because propagation lengths become shorter, or $q^{\prime\prime}$ increases, as ${E}_{F}$ decreases. In Fig. 4c, $q^{\prime\prime}$ plotted in polar coordinates is the imaginary counterpart of the IFC referred to as the loss contour. Loss contour calculations consider finite thickness α-MoO_3_ with intrinsic material scattering rate *γ* = 4 cm^−1^ and intraband-only graphene with doping-independent scattering rate ${\gamma }_{g}=40$ cm^−1^. Loss contours show $q\hbox{''}$ increasing along all directions as ${E}_{F}$ decreases, consistent with finite-difference time-domain simulations (Supplementary Fig. 8). The exact ${E}_{F}$ at which [001] modes are first observable, initiating this practical topological transition, will thus depend on the spatial resolution and the thickness of the α-MoO_3_ slab (Supplementary Fig. 9). Additionally, at very low doping, plasmons pass the interband transition threshold and are overdamped^29^. Nonlocal and other corrections to the graphene conductivity will also become important^30^.

Loss contours at intermediate doping (e.g., solid blue line in Fig. 4c) reveal that $q\hbox{''}$ decreases as the wavevector rotates away from the [001] direction (imagine black arrow rotating with length matching the loss contour). Dissipation can thus be reduced relative to unhybridized graphene (dashed blue line) by changing propagation direction since $\gamma \, < \, {\gamma }_{g}$. The same holds for the scattering rate $\Gamma ={v}_{g}q^{\prime\prime}$, where ${v}_{g}$ is the group velocity (Fig. 4d). The scattering rate in the two-dimensional approximation (red line in Fig. 1i) provides some intuition on where the excess dissipation goes. Recall that the α-MoO_3_ phonon splits the graphene plasmon into two modes ${\omega }_{+}$ and ${\omega }_{-}$ with respective scattering rates Γ_+_ and Γ_−_. As the wavevector angle $\theta$ rotates in Fig. 1i, the ${\omega }_{+}$ loss contour (red line) moves from stronger coupling where ${\Gamma }_{+} < {\Gamma }_{-}$, through a degeneracy where ${\omega }_{+}$ and ${\omega }_{-}$ share losses equally, to weaker coupling where ${\Gamma }_{+} \, > \, {\Gamma }_{-}$. The ${\omega }_{-}$ loss follows the opposing path (pink line) with ${\Gamma }_{-}$ increasing when ${\Gamma }_{+}$ decreases and vice versa such that ${\Gamma }_{+}+{\Gamma }_{-}={\gamma }_{g}+\gamma$ for fixed ${\gamma }_{g}$ and $\gamma$.

In summary, we have demonstrated an electronically-induced topological transition and the existence of SP^3^/HP^3^ hybrid modes in a doped graphene/α-MoO_3_ heterostructure by direct imaging of polaritons using an s-SNOM. Our observations are consistent with full calculations for the electrodynamics of coupled plasmon-phonon modes. Also, we demonstrated the tunability of hybrid polariton modes by modifying doping level and excitation frequency. Further, our nano-imaging data augmented with calculations reveal an intimate relationship between plasmon-phonon coupling and the properties of hybridized hyperbolic polaritons. Graphene/α-MoO_3_ integrated into a gated structure will serve as a nanophotonic platform for engineering devices with dynamical isofrequency topologies and directional plasmon-phonon coupling. Furthermore, patterning WO_x_/graphene on α-MoO_3_ could inspire new forms of photonic crystals with subwavelength periodic changes in isofrequency topology. Also, combining a graphene/α-MoO_3_ device with an ultrafast laser may permit picosecond^31^ switching of isofrequency topology for time-varying metasurfaces.

*Final note:* the authors became aware of the following relevant works by Álvarez-Pérez et al.^32^; Bapat et al.^33^; Hu et al.^34^; and Zeng et al.^35^ after completion of this work.

## Methods

### Sample fabrication

High-quality WO_x_-doped graphene/α-MoO_3_ heterostructures were fabricated using a polycaprolactone polymer-based dry transfer technique^36^. First, we exfoliate α-MoO_3_ bulk crystals purchased from a materials supplier (HQ Graphene) onto SiO_2_/Si substrates. A monolayer or bilayer WSe_2_ on graphene heterostructure is then transferred onto a predetermined α-MoO_3_ flake. Finally, we treat samples in a UV-ozone generator (Jelight UVO-cleaner) at 300 K to oxidize the topmost WSe_2_ monolayer into WO_x_^37^.

### Scanning near-field optical microscopy

A Neaspec neaSNOM near-field microscope was used with a DRS Daylight Solutions Hedgehog tunable quantum cascade laser TLS-SK-41112-HHG (λ=10.86–11.67 µm). Standard PtIr-coated Arrow tips with 75 kHz resonance frequencies were used with tapping amplitudes of about 40 nm in contact. The signal localized under the apex of the tip is isolated in the backscattered signal by demodulation at the 1st−5th tip tapping harmonics in a pseudoheterodyne detection scheme^38^.

### Electrodynamics simulations

Simulations of the electric field distributions on graphene/MoO_3_ heterostructures were performed with the Ansys Lumerical finite difference time domain (FDTD) solver for Maxwell’s equations (https://www.lumerical.com/products/fdtd/). We used a point dipole source positioned 20 nm above the top surface of the graphene/MoO_3_ structure and polarized along the z direction. Electric field monitors were placed on the top surface. The boundary conditions were set to the perfectly matched layer option. The NumPy fast Fourier transform library was used to compute isofrequency contours from FDTD wavefronts. In particular, we used the fft2 function followed by the fftshift function to set the zero-frequency component to the center.

### Data fitting procedure

To extract polariton momenta $q^{\prime}$ and dissipation $q^{\prime\prime}$ from near-field amplitude images, it is necessary to fit a damped oscillatory function to line profiles. In this work, we use a zeroth-order Hankel function of the first kind ${H}_{0}^{1}$ of the following form:

M1 $${S}_{A}=\left|A{H}_{0}^{1}\left(2\left({q}^{\prime}-i{q}^{^{\prime\prime} }\right)\left(x-{x}_{0}\right)\right)\right|+B(x-{x}_{0})+C,$$

where the fitting parameters are $A,B,C,q^{\prime}$ and ${q}^{^{\prime\prime} }$. The factor of two in the argument of the Hankel function comes from the fact that we are observing interference fringes between tip-launched and edge-reflected polaritons. The ${x}_{0}$ value was fixed to match the position of the first peak, which was then cut off during fitting so that contributions from higher-order modes may be neglected.

### Raman spectroscopy

Raman measurements were conducted on a commercial Bruker Senterra system using an excitation wavelength of 532 nm at 2 mW with a 60 second integration time. A grating of 1200 line/mm was used to attain an energy resolution of 3–5 cm^−1^.

## Supplementary information

Supplementary Information

## Data Availability

Relevant data supporting the key findings of this study are available within the article and the Supplementary Information file. All raw data generated during the current study are available from the corresponding authors upon request.
