# Supplementary material for: Surface plasmons induce topological transition in graphene/α-MoO3 heterostructures
Source: Nat Commun. 2022 Jun 28;13:3719. doi: 10.1038/s41467-022-31477-z (PMC9240047; doi:10.1038/s41467-022-31477-z)
Supplement: Supplementary file 1 — Supplementary Information [file 41467_2022_31477_MOESM1_ESM.pdf]

Supplementary Materials for

Surface plasmons induce topological transition in graphene/α-MoO_3 heterostructures

Francesco L. Ruta†[1,2], Brian S. Y. Kim†[3], Zhiyuan Sun[1], Daniel J. Rizzo[1], Alexander S. McLeod[1], Anjaly Rajendran[3], Song Liu[3], Andrew J. Millis[1,4], James C. Hone[3], D. N. Basov*[1]*

Correspond to: *f.ruta@columbia.edu, *db3056@columbia.edu

Table of Contents **pg(s)**

Supplementary Figures 1-9

1. *Graphene/α-MoO_3 heterostructure schematic* 2
2. *Raman spectroscopy on graphene/α-MoO_3 heterostructures with varying WSe_2 layer number* 6
3. *Rescaled near-field image of circular void and collocated topography* 8
4. *Undoped graphene/α-MoO_3 heterostructure (no WO_x)* 8
5. *Topography and frequency-dependent near-field imaging* 9
6. *Comparison of [001] and 8°∠[001] plasmon-phonon polariton dispersions* 10
7. *Effect of WSe_2 layer on graphene/α-MoO_3 polariton dispersion* 10
8. *Polariton wavefront near topological transition* 11
9. *Dependence of hybrid polariton momentum on α-MoO_3 thickness* 12

Supplementary Notes 1-3

1 **Polariton dispersion in graphene/α-MoO_3 heterostructures** 2-4
   a. *Arbitrary thickness slab*
   b. *Semi-infinite thickness limit*
   c. *Two-dimensional limit*
   d. *Complex root finding algorithm*

2 **Coupled plasmons and in-plane hyperbolic phonons** 5-6
   a. *Derivation of Equation 1*
   b. *Hybrid mode scattering rate*

3 **Doping level of WO_x/graphene/α-MoO_3 interfaces** 7-11
   a. *Extracting doping level from near-field data*
   b. *Raman spectroscopy*
   c. *Samples without WO_x*
   d. *Effect of WSe_2 layer on optical properties*

Supplementary References 1-13 13

Supplementary Note 1: Polariton dispersion in graphene/α-MoO_3 heterostructures

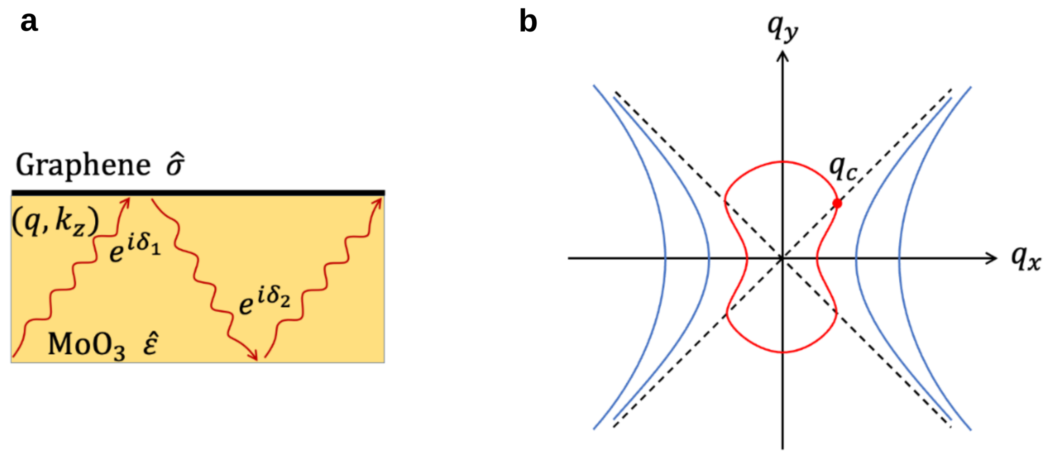

Supplementary Figure 1: Graphene/α-MoO_3 heterostructure schematic. a, Schematic of the graphene/α-MoO_3 device. Wavey lines are waveguide modes with the reflection phase shifts shown. **b**, schematic isofrequency contours of polariton modes of the device.

a. Arbitrary thickness slab

The device is shown schematically in Supplementary Figure 1a. The polariton modes are described by quasistatic electric potentials: $\varphi = A_1 e^{i(qr+k_z z)} + A_2 e^{i(qr-k_z z)}$ inside α-MoO_3 and $\varphi = A_3 e^{i(qr-k_{z,0} z)}$ in the space above the graphene layer, where $q = (q_x, q_y)$ is the in-plane wavevector. Given fixed frequency ω, we need to determine the in-plane momenta q of the polaritons. The quasistatic Maxwell's equations inside α-MoO_3, $\varepsilon_{ij}\partial_i\partial_j\varphi = 0$, lead to the bulk dispersion relation

$$\varepsilon_x q_x^2 + \varepsilon_y q_y^2 + \varepsilon_z k_z^2 = 0. \qquad \text{(S1)}$$

The boundary conditions for φ on the top and bottom surfaces of α-MoO_3 give two equations satisfied by $A_{1,2}$, which can be interpreted as two complex reflection "phase shifts":

$$\frac{A_2}{A_1} = e^{i\delta_1}, \qquad \frac{A_1 e^{-ik_z d}}{A_2 e^{ik_z d}} = e^{i\delta_2}. \qquad \text{(S2)}$$

Solving the boundary condition equation on the top and bottom interfaces, one obtains the well-known expression for the complex phase shift[1,2]:

$$e^{i\delta_i} = \frac{1 - i\frac{\varepsilon_{\mathrm{sub}}}{\sqrt{-\varepsilon_\parallel \varepsilon_z}} + \frac{4\pi q \sigma_g}{\omega\sqrt{-\varepsilon_\parallel \varepsilon_z}}}{1 + i\frac{\varepsilon_{\mathrm{sub}}}{\sqrt{-\varepsilon_\parallel \varepsilon_z}} - \frac{4\pi q \sigma_g}{\omega\sqrt{-\varepsilon_\parallel \varepsilon_z}}}, \qquad \text{(S3)}$$

where $\varepsilon_{\mathrm{sub}}$ is the permittivity of the substrate under α-MoO3, σ_g is the two-dimensional sheet conductivity of the top interface (if there is any 2D conducting layer there), and ε_z, $\varepsilon_\parallel$ are the complex dielectric permittivities of the slab. We have defined $\varepsilon_\parallel = \varepsilon_x \cos^2\theta + \varepsilon_y \sin^2\theta$ as the effective in-plane dielectric permittivity of α-MoO3 where θ is the angle between q and the x direction. Note that δ_i are not necessarily real numbers and can be imaginary for evanescent waves. Existence of solutions to Equation S2 leads to the polariton eigenmode condition in the near-field limit:

$$2dk_z + \delta_1 + \delta_2 = 2\pi N, \tag{S4}$$

where $N \in \mathbb{N}_0$ is the mode order. Graphene affects the mode via its effect on the phase shift δ_1, which leads to hybridization between graphene plasmons and α-MoO3 phonon polaritons. Equations S1, S3, and S4 determine the isofrequency contours (IFCs) in Supplementary Figure 1b for $\varepsilon_x < 0, \varepsilon_y > 0, \varepsilon_z > 0$, which happens in the experimental frequency range. Combining Equations S1, S3, and S4 gives:

$$2dq\sqrt{\frac{-\varepsilon_\parallel}{\varepsilon_z}} - i\ln\left(\frac{1-\frac{i}{\sqrt{-\varepsilon_\parallel\varepsilon_z}}+\frac{4\pi\sigma_g q}{\omega\sqrt{-\varepsilon_\parallel\varepsilon_z}}}{1+\frac{i}{\sqrt{-\varepsilon_\parallel\varepsilon_z}}-\frac{4\pi\sigma_g q}{\omega\sqrt{-\varepsilon_\parallel\varepsilon_z}}}\right) - i\ln\left(\frac{1-i\frac{\varepsilon_{sub}}{\sqrt{-\varepsilon_\parallel\varepsilon_z}}}{1+i\frac{\varepsilon_{sub}}{\sqrt{-\varepsilon_\parallel\varepsilon_z}}}\right) = 2\pi N. \tag{S5}$$

The first-order hybrid mode ($N = 0$) crosses over from a hyperbolic plasmon-phonon polariton (HP[3]) for $\theta < \theta_c$ to a surface plasmon-phonon polariton (SP[3]) for $\theta > \theta_c$ where the critical angle θ_c is defined such that $\mathrm{Re}\,\varepsilon_\parallel(\theta_c) = 0$. The critical wavevector $q_c = q(\theta_c)$ can be computed analytically when $k_z \to 0$ (assuming lossless), $\delta_1 \to \pi$, and $\delta_2 \to -\pi$. With these assumptions, Equation S5 simplifies to

$$\frac{dq_c}{\varepsilon_z} - \frac{1}{\frac{2q_c}{q_p}-1} + \frac{1}{\varepsilon_{\mathrm{sub}}} = 0, \tag{S6}$$

where $q_p \equiv \frac{i\omega}{2\pi\sigma_g}$, and the dielectric constant of air has been replaced by unity. Solving Equation S6 gives

$$\frac{q_c}{q_p} = \frac{1}{4}\left(1 - \zeta + \sqrt{(1-\zeta)^2 + 8\zeta}\right), \qquad \zeta \equiv \frac{2\varepsilon_z}{dq_p} \tag{S7}$$

In the limit of charge neutrality ($q_p \to \infty$), the value of q_c diverges. This is the asymptote of the unbounded hyperbolic IFC when there is no doping. As soon as $\sigma_g \neq 0$, q_c becomes finite. We know also q_y ($\theta = 90°$) corresponds to an unhybridized graphene plasmon: q_y likewise will only diverge when $\sigma_g = 0$. We assume then that q between $\theta_c \leq \theta \leq 90°$ all diverge together, and we track the value of q_c to check the boundedness of the IFC. Bounded and unbounded IFCs are not homeomorphic and thus we say that a topological transition occurs upon incremental doping away from $\sigma_g = 0$. This topological transition admits discontinuous changes in the polaritonic density of states, analogously to the Lifshitz transition for Fermi surfaces[3].

b. Semi-infinite thickness limit

In the thick limit, $dq_p \gg 2\varepsilon_z$, we have $x \ll 1$ and $q/q_p = 1/2$ at the critical angle. At $\theta = \frac{\pi}{2}$, we have $q/q_p = \frac{1+\sqrt{\varepsilon_y \varepsilon_z}}{2}$. For $\theta_c < \theta < \frac{\pi}{2}$, we have $q/q_p = \frac{1+\sqrt{\varepsilon_\perp(\theta)\varepsilon_z}}{2}$.

c. Two-dimensional limit

In certain limits, the whole device can be viewed as a two-dimensional plane with net sheet conductivity $\hat{\sigma} = \hat{\sigma}_{\mathrm{g}} + \hat{\sigma}_{\mathrm{MoO_3}}$ with contributions from both graphene and α-MoO_3, where $\hat{\sigma}_{\mathrm{MoO_3}} = \frac{d\omega}{4\pi i}(\varepsilon - 1)$ is related to the in-plane components of the dielectric tensor of α-MoO_3. Hybrid polaritons can be viewed as the plasmonic modes of the 2D plane, whose dispersions are determined by

$$\varepsilon_{\mathrm{2D}}(\omega, q) = 1 + V_q \frac{i}{\omega} q_i \sigma_{ij} q_j = 0 \rightarrow \frac{-2\pi i}{\omega}\left(\sigma_x \cos^2\theta + \sigma_y \sin^2\theta\right) = \frac{1}{q}. \quad \text{(S8)}$$

Therefore, the dispersion is in-plane hyperbolic if $\operatorname{Im}\sigma_x$ and $\operatorname{Im}\sigma_y$ are opposite in sign. By increasing the doping level of graphene, one can obviously render them the same sign, thus changing the polaritons to non-hyperbolic. However, the 2D limit makes the approximation that the in-plane electric field is independent on z inside the α-MoO_3 slab, which is generally not true. Note that there is no HP^3 mode in the 2D limit. Hybrid polaritons in the strong coupling regime are always SP^3 and the hyperbolic α-MoO_3 phonon is effectively a surface phonon at ω_{TO}.

d. Complex root finding algorithm

Equation S5 must be solved numerically when $\sigma_g \neq 0$ since the polariton q appears in the phase shift term. We search for roots using the LMFIT[4] wrapper for scipy.optimize methods in Python with the Nelder-Mead simplex algorithm. Since logarithms are multivalued for complex arguments, one must be careful when choosing how to represent the multivalued function. Branch cuts can cause problems for local root-finding algorithms like Nelder-Mead and indeed do cause our algorithm to yield unusual results for the imaginary part of q at intermediate θ. To improve the stability of the root finding algorithm, we do not search for roots of Equation S5 directly, but rather of the pointwise product of its first several Riemann sheets[5].

Supplementary Note 2: Coupled plasmons and in-plane hyperbolic phonons

a. Derivation of Equation 1

Supposing the Fermi energy $|E_F| \gg kT$, the graphene optical conductivity σ_g computed with the Kubo formula using the bare bubble approximation in Gaussian units (m/s) is given by[6]:

$$\sigma_g = \frac{e^2}{8\pi^3\hbar^2\varepsilon_0}\frac{|E_F|}{\gamma_g - i\omega}, \tag{S9}$$

where γ_g is the scattering rate of graphene. Only intraband contributions are considered and nonlocal effects are neglected since $q \ll k_F$. The dielectric tensor components of α-MoO_3 are modeled by the TO-LO equation[7] with a single oscillator along each crystallographic direction:

$$\varepsilon = \varepsilon_\infty\left(1 + \frac{\omega_{LO}^2 - \omega_{TO}^2}{\omega_{TO}^2 - \omega^2 - i\omega\gamma}\right), \tag{S10}$$

where ω_{TO} and ω_{LO} are the frequencies of the transverse and longitudinal optical phonon modes, respectively. In the middle reststrahlen band, only the x-direction or [100] phonon is strongly dispersive, so we set the y-direction permittivity to a constant value for simplicity. By combining Equations S8-S10, we can then write:

$$\varepsilon_{2\mathrm{D}}(\omega, q) = 1 - \frac{\omega_{pl}^2(q)}{\omega(\omega + i\gamma_g)} + \frac{qd}{2}\left\{\left(\varepsilon_x^\infty \frac{\omega_{LO}^2 - \omega_{TO}^2}{\omega_{TO}^2 - \omega^2 - i\omega\gamma_x} + \varepsilon_x^\infty - 1\right)\cos^2\theta + \left(\varepsilon_y - 1\right)\sin^2\theta\right\}, \tag{S11}$$

where $\omega_{pl}^2(q) \equiv \frac{q|E_F|e^2}{2\pi\hbar^2\varepsilon_0}$. Finally, taking $\varepsilon_y = 1$ and $\varepsilon_x^\infty = 1$, this simplifies to Equation 1 in the main text:

$$\varepsilon_{2D}(\omega, q) = 1 - \frac{\omega_{pl}^2(q)}{\omega^2 + i\omega\gamma_g} + \alpha(q,\theta)\frac{\omega_{LO}^2 - \omega_{TO}^2}{\omega_{TO}^2 - \omega^2 - i\omega\gamma}, \tag{S12}$$

with $\alpha(q,\theta) \equiv \frac{qd}{2}\cos^2\theta$ serving as a "coupling strength" parameter. Setting Equation S12 to zero, the complex ω corresponding to the self-sustained polariton modes were computed analytically in symbolic mathematical software.

b. Hybrid mode scattering rate

From Equation S8, we can also obtain an approximate semi-analytical equation for the direction-dependent scattering rate in the two-dimensional limit. The scattering rate of the hybrid mode Γ can be obtained self-consistently by satisfying the following relation:

$$\frac{\gamma_g - \Gamma}{\Gamma - \gamma} \approx \frac{\left(\omega^2 + \frac{\Gamma^2}{4}\right)\left(\omega^2 + \left(\gamma_g - \frac{\Gamma}{2}\right)^2\right)}{\omega^2(\Gamma - \gamma)^2 + \left(\omega^2 - \frac{\Gamma}{2}\left(\frac{\Gamma}{2} - \gamma\right) - \omega_{TO}^2\right)^2}\Upsilon(E_F)\cos^2\theta, \tag{S13}$$

where $\Upsilon(E_F) \equiv 2\pi^2\hbar^2\varepsilon_0 d(\omega_{LO}^2 - \omega_{TO}^2)/(e^2|E_F|) > 0$. When $\theta = 90°$, the righthand side goes to zero, forcing $\Gamma = \gamma_g$ on the lefthand side. In contrast, at $\theta = 0°$, Γ is not forced to equal the phonon scattering rate. Since the righthand side is always positive, it must be the case that $\gamma \leq \Gamma(\theta) \leq \gamma_g$ given that $\gamma_g > \gamma$. Numerical solutions to Equation S13 are plotted in Figure 4d in the main text.

To obtain the hybrid mode scattering rate $\Gamma = v_g q''$ for a finite thickness slab, we can compute $q''(\omega)$ and $q'(\omega \pm h)$ numerically using Equation S5 and then use a central difference formula with $h = 10^{-9}$ cm^{-1} to estimate the group velocity:

$$v_g \approx \frac{2h}{q'(\omega+h) - q'(\omega-h)} \tag{S14}$$

We remark that solutions obtained through Equations S13 and S14 are qualitatively consistent, but Equation S14 can be unstable at intermediate angles.

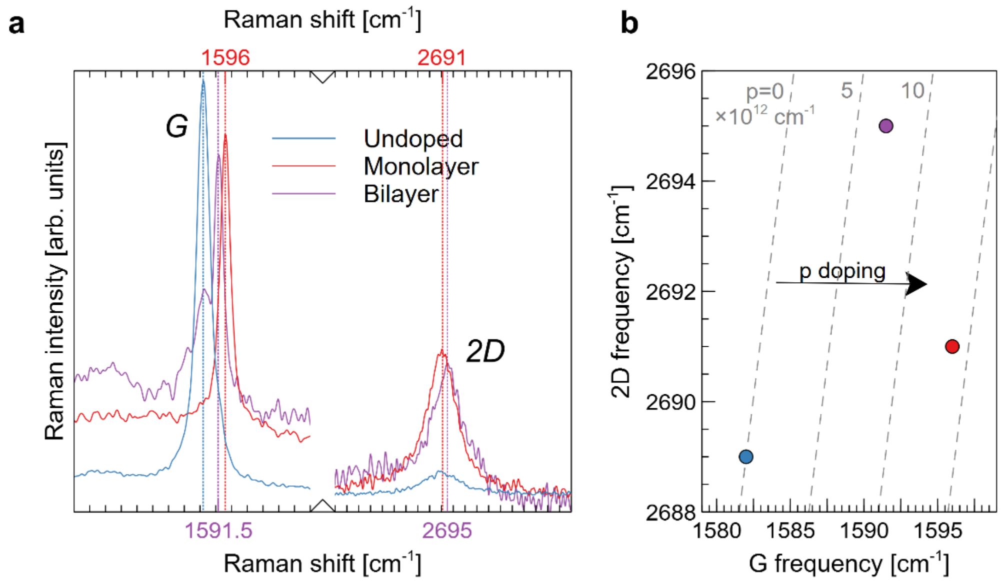

Supplementary Figure 2: Raman spectroscopy on graphene/α-MoO_3 heterostructures with varying WSe_2 layer number. a, *G* and *2D* peaks in Raman spectra of graphene/α-MoO_3 heterostructures with 0 (blue), 1 (red), and 2 (purple) layers of tungsten diselenide (WSe_2) stacked on top and oxidized. **b**, the oxidized WSe_2 hole dopes graphene by a charge-transfer process, as evidenced by the shifted *G* and *2D* Raman peaks. Since oxidation is self-limited, only the topmost layer is oxidized. The pristine bottom WSe_2 layer in the bilayer structure acts as a spacer that reduces the doping level. Also, the bilayer *G* peak has a secondary undoped peak due to the many bubbles and tears in the bilayer sample.

Supplementary Note 3: Doping level of WO_x/graphene/α-MoO_3 interfaces

a. Extracting doping level from near-field data

Experimental Fermi energies E_F reported in the main text for WO_x/graphene/α-MoO_3 (monolayer) and WO_x/1L-WSe_2/graphene/α-MoO_3 (bilayer) samples were determined by fitting polariton dispersions to energy-momentum (ω, q) data with the E_F of graphene as a free parameter. In other words, we solve the following nonlinear least squares problem assuming homoscedasticity:

$$\hat{E}_F = \underset{E_F}{\operatorname{argmin}} \sum_i \big(q(\omega_i, E_F) - q_{obs}(\omega_i)\big)^2 \tag{S15}$$

We report E_F to the nearest 0.05 eV, since likelihood-based 95% confidence intervals for this type of estimation were previously determined to be about ± 0.03 eV[8]. The best-fit dispersion for the 0.45 eV sample is shown in Figure 3e in the main text. Similar dispersions were obtained for the 0.60 eV samples.

b. Raman spectroscopy

To corroborate the doping levels extracted from fitting polariton dispersions, we also performed Raman spectroscopy on these samples. Supplementary Figure 2a shows Raman spectra on monolayer, bilayer, and undoped samples. The undoped graphene *G* and *2D* peaks appear at 1582 cm^{-1} and 2689 cm^{-1}, respectively. The bilayer *G* peak shifts to 1591.5 cm^{-1} and the monolayer *G* peak shifts further to 1596 cm^{-1} while *2D* peaks shift to 2695 cm^{-1} and 2691 cm^{-1} for bilayer and monolayer samples, respectively. The gray lines in Supplementary Figure 2b represent the *G* and *2D* peak positions corresponding to approximate doping levels from a standard analysis[9] using empirical parameters from Supplementary Reference 10. The undoped sample was used as a zero-carrier reference point. Qualitatively, our analysis agrees with trends determined from dispersion fitting.

Additionally, the bilayer sample has a secondary undoped *G* peak likely corresponding to the many bubbles and tears seen in topography (Supplementary Figure 5a). Finally, we remark that the spectral weight ratio of *G* to *2D* peaks changes visibly upon doping. Overall, Raman spectroscopy is consistent with the appearance of plasmon polaritons in near-field images from substantial doping of the graphene layer.

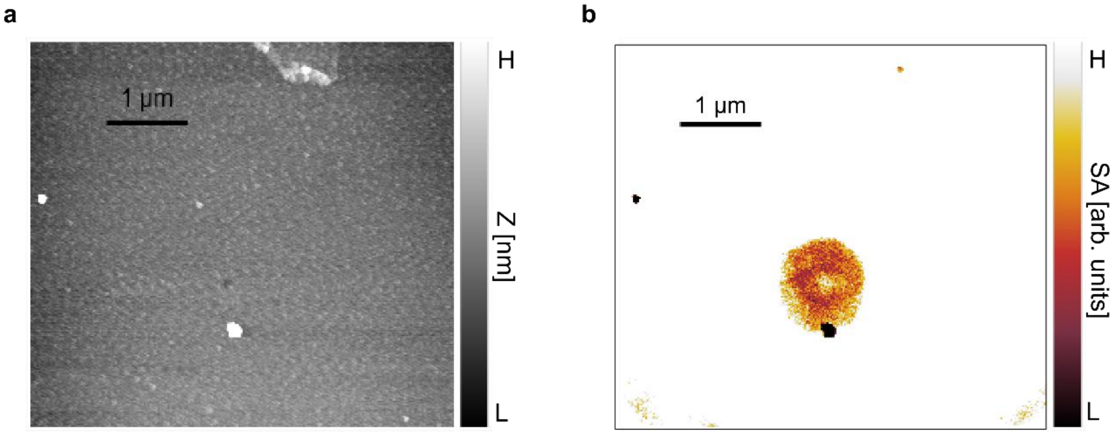

Supplementary Figure 3: Rescaled near-field image of circular void and collocated topography. a, topography image collocated with Figure 2a in the main text. The circular void does not appear in topography, suggesting that the conductivity boundary seen in **b** is caused by a region of unoxidized WSe_2. The circular region was likely shielded from oxidation by nearby dirt particles. **b**, Figure 2a with a rescaled color scale, highlighting the undoped phonon polaritonic cavity inside the circular void.

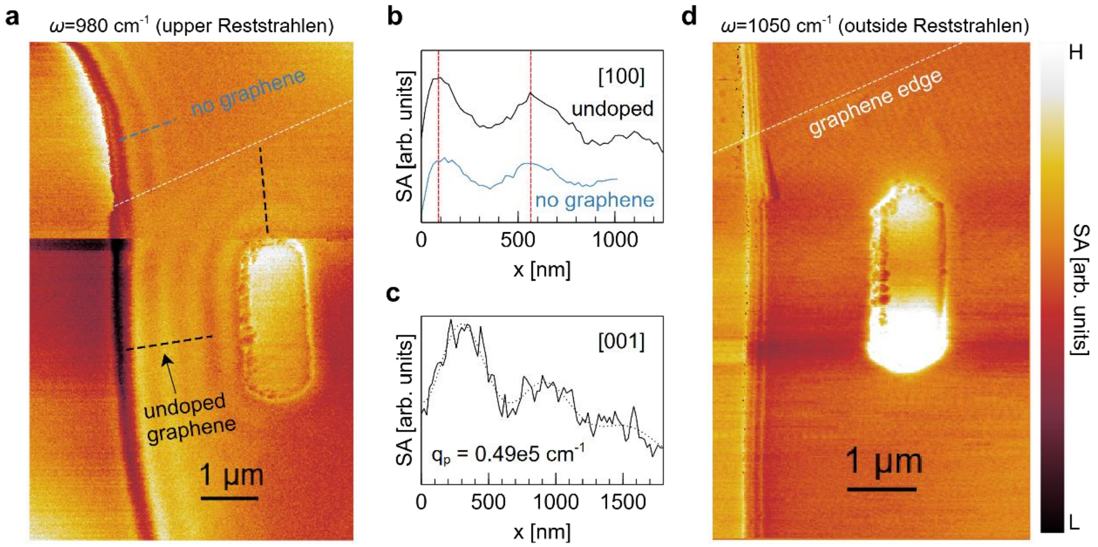

Supplementary Figure 4: Undoped graphene/α-MoO_3 heterostructure (no WO_x). a, near-field amplitude image at laser frequency ω=980 cm^{-1} of an undoped graphene/α-MoO_3 heterostructure with a gold antenna deposited by electron beam lithography. **b**, black and blue profiles correspond to black and blue dashed lines in **a** on and off graphene, respectively. The graphene edge is indicated by the white dashed line. There is no significant difference in polariton momentum (look at red dashed lines), suggesting that there is little to no work-function-mediated doping of graphene on crystalline α-MoO_3. **c**, the out-of-plane hyperbolic upper reststrahlen band [001] polariton mode is also consistent with little to no doping. **d,** plasmons are not visible at ω=1050 cm^{-1}, where there are no phonon hybridization effects. Also, the graphene-covered region is not brighter than bare α-MoO_3.

c. Samples without WO_x

In this section, we present an extended dataset for an undoped graphene/α-MoO_3 heterostructure without WO_x. We show that this data is consistent with low to no doping, that is, a Fermi energy in graphene approximately at charge neutrality. This is in contrast to Raman data on graphene on amorphous, oxygen-deficient MoO_{3-x} heterostructures showing evidence of hole-doping graphene to ~0.28 eV from charge transfer between constituent layers[11].

In Supplementary Figure 4a, we show a near-field image of a ~200 nm α-MoO_3 flake with graphene but without WO_x. The edge of the graphene sheet is marked with a white dashed line. Two representative line profiles are taken on and off the graphene (black and blue dashed lines, respectively). Here, we look at the upper reststrahlen band of α-MoO_3, which is out-of-plane hyperbolic with propagation allowed along all in-plane directions. The wavelengths of the [100] phonon polariton fringes shown in the line profiles (Supplementary Figure 4b) are not significantly different on and off graphene, implying that there is no observable plasmon-phonon hybridization. Likewise, the [001] polariton fringes are observed (Supplementary Figure 4c) with momentum consistent with no hybridization. Furthermore, at 1050 cm^{-1}, outside of any α-MoO_3 reststrahlen band, where plasmons should appear unimpeded by phonon hybridization effects, we do not see plasmonic fringes (Supplementary Figure 4d).

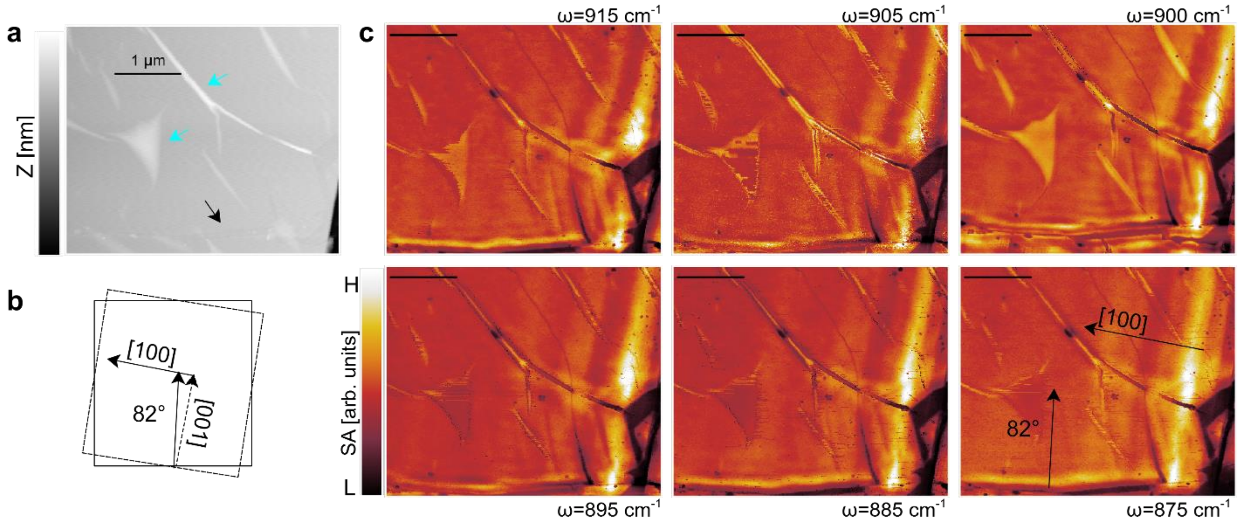

Supplementary Figure 5: Topography and frequency-dependent near-field imaging. a, topography of WO_x/WSe_2/graphene/α-MoO_3 stack collocated with near-field images. Cyan arrows point to bubbles (raised topography) and black arrow points to a physical edge of WO_X/graphene. **b**, orientation of α-MoO_3 flake relative to graphene edge. The normal to the graphene edge where line profiles were extracted is 82° from the [100] direction, or 8° from the [001] direction. **c**, frequency-dependent fourth-harmonic near-field imaging showing dispersion of hybrid modes from 875-915 cm^{-1}. All scale bars are 1 μm.

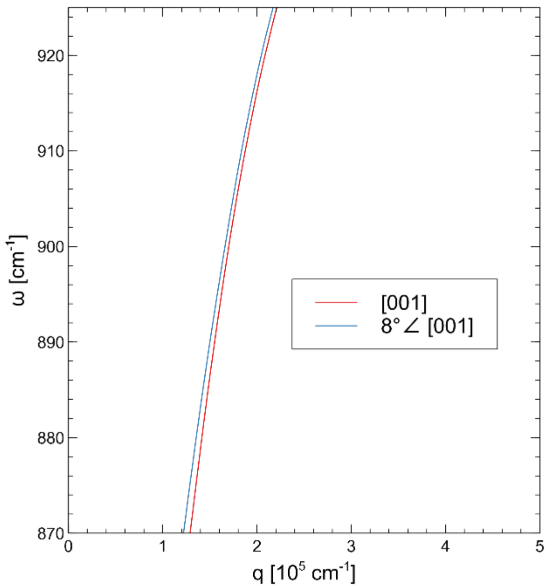

Supplementary Figure 6: Comparison of [001] and 8°∠ [001] plasmon-phonon polariton dispersions. The physical boundary of graphene in Figs. 3b and S5 is 8° off from the crystal axis of α-MoO_3. Modes reflected from this boundary propagate along $\theta = 82°$ or $8°\angle$ [001]. Momenta of these modes are practically indistinguishable from [001] modes at the investigated excitation frequencies, as shown in dispersion calculation above. We thus neglect the off-angle correction in the main text.

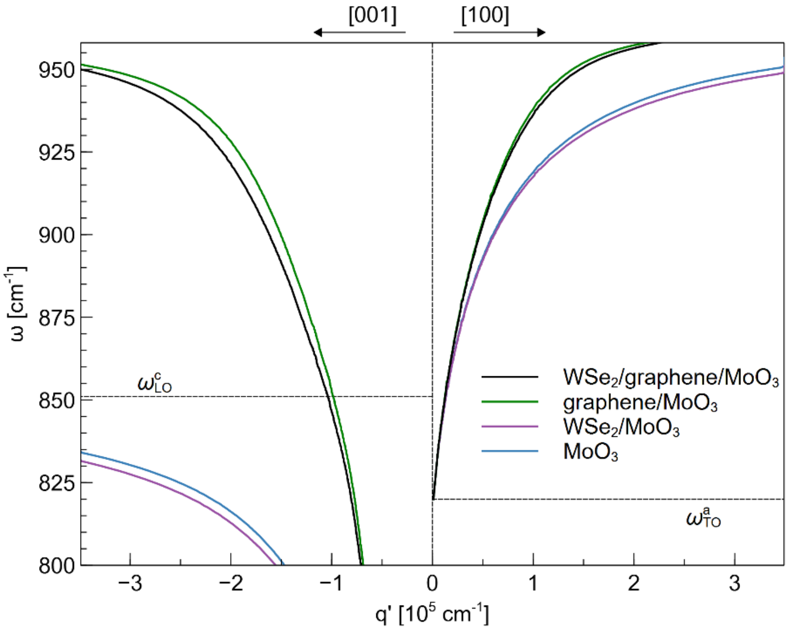

Supplementary Figure 7: Effect of WSe_2 layer on graphene/α-MoO_3 polariton dispersion. The high dielectric permittivity of WSe_2 leads to increased confinement of both [100] and [001] modes (compare black and green lines or purple and blue lines). However, without doped graphene, the WSe_2 layer alone cannot induce a topological transition at the frequencies studied in this work (note that purple line cannot exceed ω_{LO}^{c} along the [001] direction without reducing confinement). $\omega_{TO(LO)}^{j}$ are TO(LO) frequencies of j-axis phonons: values plotted as black dashed lines.

d. Effect of WSe_2 layer on optical properties

A 1.4 nm monolayer of pristine WSe_2 was included in Figure 3e calculations in the main text. The mid-infrared dielectric tensor of bulk WSe_2 from Supplementary Reference 12 was used in calculations. We assume the topmost, oxidized WO_x layer has a dielectric permittivity of unity. A thin layer of WSe_2 on a polaritonic medium has a significant effect on the confinement of polariton modes due its high dielectric permittivity[13], but it cannot induce a topological transition of α-MoO_3 phonon polaritons without doped graphene. Supplementary Figure 7 shows the calculated dispersions with and without WSe_2 both with and without graphene. The WSe_2 layer can increase confinement of α-MoO_3 modes, but will not allow them to surpass ω_{LO}^{c} and initiate a topological transition. Lastly, note that Figure 2f in the main text shows that [100] modes are actually less confined on WO_x, implying that hybridization with graphene plasmons is the dominant effect on α-MoO_3 phonon polaritons.

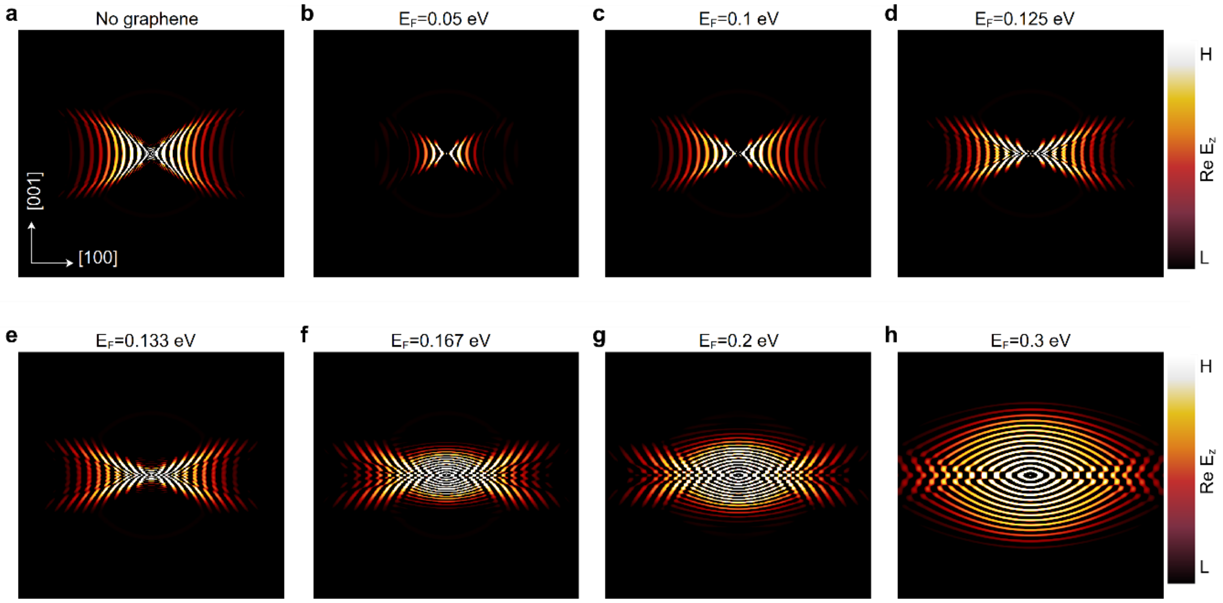

Supplementary Figure 8: Polariton wavefront near topological transition. a-h, Simulated Re E_z field on a 100 nm α-MoO_3 slab with graphene of various Fermi energies E_F under ω=926 cm^{-1} illumination. Polariton wavefronts evolve from hyperbolic to ovular with increased doping. [001] modes first become visible around E_F=0.125 eV, **d**, for this set of simulation parameters. At lower dopings, **b** and **c**, [001] modes are overdamped and/or too highly confined to observe for the selected mesh resolution.

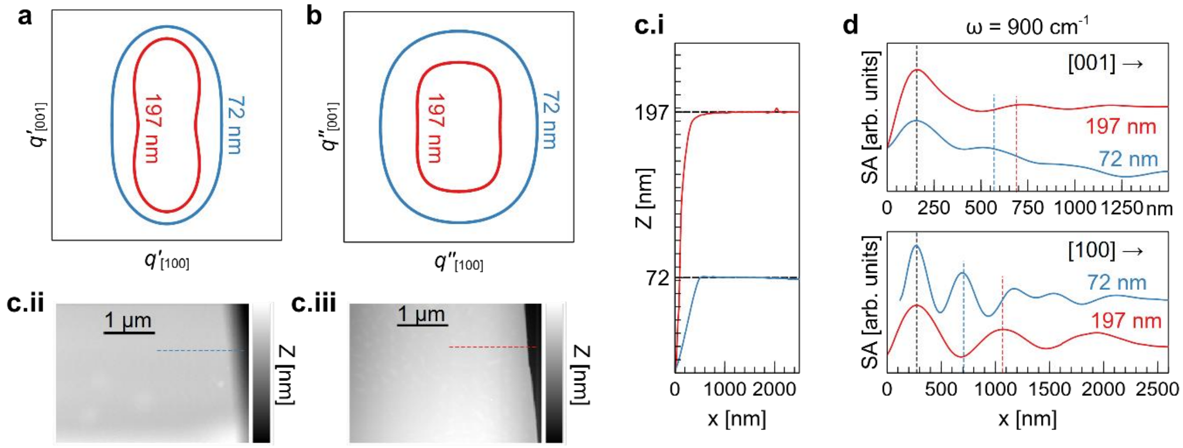

Supplementary Figure 9: Dependence of hybrid polariton momentum on α-MoO_3 thickness. a, isofrequency and, **b**, loss contours of graphene/α-MoO_3 heterostructures with 72 nm (blue) and 197 nm (red) thick α-MoO_3 slabs. Both momentum q' and dissipation q'' increase with decreasing α-MoO_3 thickness. **c**, experimental topography line profiles (i) taken from corresponding atomic force microscopy images (ii and iii). **d**, experimental near-field amplitude line profiles on structures with E_F=0.60 eV and varying thickness (corresponding near-field images shown in Figures 2a (197 nm) and 3a (72 nm) in the main text). Note that both [001] and [100] mode wavelengths decrease in 72 nm sample (blue) relative to 197 nm sample (red). *Nota bene*: 0.60 eV line profiles in Figure 3c are from Figure 2a, not Figure 3a.

Supplementary References

[1] Z. Sun, Á. Gutiérrez-Rubio, D.N. Basov, M.M. Fogler, Hamiltonian optics of hyperbolic polaritons in nanogranules, *Nano Letters* **15**, 4455–4460 (2015)

[2] J.-S. Wu, D.N. Basov, M.M. Fogler, Topological insulators are tunable waveguides for hyperbolic polaritons, *Physical Review B* **92**, 205430 (2015)

[3] I.M. Lifshitz, Anomalies of electron characteristics of a metal in the high pressure region, *Soviet Physics JETP* **11**, 1130-1135 (1960)

[4] M. Newville, T. Stensitzki, D.B. Allen, A. Ingargiola, LMFIT: Non-linear least-square minimization and curve-fitting for Python (2014) dx.doi.org/10.5281/zenodo.11813 (accessed March 21, 2020)

[5] P. Kowalczyk, On root finding algorithms for complex functions with branch cuts, *Journal of Computational and Applied Mathematics* **314**, 1-9 (2017)

[6] P.A.D. Gonçalves, N.M.R. Peres, An Introduction to Graphene Plasmonics, Singapore: World Scientific, pg. 25 (2016)

[7] F. Gervais, B. Pirou, Anharmonicity in several-polar-mode crystals: adjusting phonon self-energy of LO and TO modes in Al_2O_3 and TiO_2 to fit infrared reflectivity, *Journal of Physics C: Solid State Physics* **7**, 2374-2386 (1974)

[8] D.J. Rizzo, *et al.*, Charge-transfer plasmon polaritons at graphene/α-$RuCl_3$ interfaces, *Nano Letters* **20**, 8438-8445 (2020)

[9] J.E. Lee, G. Ahn, J. Shim, Y.S. Lee, S. Ryu, Optical separation of mechanical strain from charge doping in graphene, *Nature Communications* **3**, 1024 (2012)

[10] M. Choi, *et al.*, High carrier mobility in graphene doped using a monolayer of tungsten oxyselenide, *Nature Electronics* **4**, 731-739 (2021)

[11] Q. Wu, *et al*., Electronic structure of MoO_{3-x}/graphene interface, *Carbon* **65**, 46-52 (2013)

[12] F.L. Ruta, A.J. Sternbach, A.B. Dieng, A.S. McLeod, D.N. Basov, Quantitative nanoinfrared spectroscopy of anisotropic van der Waals materials, *Nano Letters* **20**, 7933-7940 (2020)

[13] A.M. Dubrovkin, B. Qiang, H.N.S. Krishnamoorthy, N.I. Zheludev, Q.J. Wang, Ultra-confined surface phonon polaritons in molecular layers of van der Waals dielectrics, *Nature Communications* **9**, 1762 (2018)
